# Can screening instruments accurately determine poor outcome risk in adults with recent onset low back pain? A systematic review and meta-analysis

**DOI:** 10.1186/s12916-016-0774-4

**Published:** 2017-01-19

**Authors:** Emma L. Karran, James H. McAuley, Adrian C. Traeger, Susan L. Hillier, Luzia Grabherr, Leslie N. Russek, G. Lorimer Moseley

**Affiliations:** 1 Sansom Institute for Health Research, University of South Australia, GPO Box 2471, Adelaide, South Australia 5001 Australia; 2 Neuroscience Research Australia, Barker Street, Randwick, Sydney, New South Wales 2031 Australia; 3 Clarkson University, 41 Elm Street, Potsdam, New York USA; 4 Prince of Wales Clinical School, University of New South Wales, High Street, Kensington, New South Wales 2052 Australia

**Keywords:** Low back pain, Screening, Prognosis, Risk, Predictive validity

## Abstract

**Background:**

Delivering efficient and effective healthcare is crucial for a condition as burdensome as low back pain (LBP). Stratified care strategies may be worthwhile, but rely on early and accurate patient screening using a valid and reliable instrument. The purpose of this study was to evaluate the performance of LBP screening instruments for determining risk of poor outcome in adults with LBP of less than 3 months duration.

**Methods:**

Medline, Embase, CINAHL, PsycINFO, PEDro, Web of Science, SciVerse SCOPUS, and Cochrane Central Register of Controlled Trials were searched from June 2014 to March 2016. Prospective cohort studies involving patients with acute and subacute LBP were included. Studies administered a prognostic screening instrument at inception and reported outcomes at least 12 weeks after screening. Two independent reviewers extracted relevant data using a standardised spreadsheet. We defined poor outcome for pain to be ≥ 3 on an 11-point numeric rating scale and poor outcome for disability to be scores of ≥ 30% disabled (on the study authors' chosen disability outcome measure).

**Results:**

We identified 18 eligible studies investigating seven instruments. Five studies investigated the STarT Back Tool: performance for discriminating pain outcomes at follow-up was ‘non-informative’ (pooled AUC = 0.59 (0.55–0.63), *n* = 1153) and ‘acceptable’ for discriminating disability outcomes (pooled AUC = 0.74 (0.66–0.82), *n* = 821). Seven studies investigated the Orebro Musculoskeletal Pain Screening Questionnaire: performance was ‘poor’ for discriminating pain outcomes (pooled AUC = 0.69 (0.62–0.76), *n* = 360), ‘acceptable’ for disability outcomes (pooled AUC = 0.75 (0.69–0.82), *n* = 512), and ‘excellent’ for absenteeism outcomes (pooled AUC = 0.83 (0.75–0.90), *n* = 243). Two studies investigated the Vermont Disability Prediction Questionnaire and four further instruments were investigated in single studies only.

**Conclusions:**

LBP screening instruments administered in primary care perform poorly at assigning higher risk scores to individuals who develop chronic pain than to those who do not. Risks of a poor disability outcome and prolonged absenteeism are likely to be estimated with greater accuracy. It is important that clinicians who use screening tools to obtain prognostic information consider the potential for misclassification of patient risk and its consequences for care decisions based on screening. However, it needs to be acknowledged that the outcomes on which we evaluated these screening instruments in some cases had a different threshold, outcome, and time period than those they were designed to predict.

**Systematic review registration:**

PROSPERO international prospective register of systematic reviews registration number CRD42015015778.

**Electronic supplementary material:**

The online version of this article (doi:10.1186/s12916-016-0774-4) contains supplementary material, which is available to authorized users.

## Background

A current trend in health service delivery towards the provision of stratified models of care [1–3] offers potential to optimise treatment benefits, reduce harms and maximise healthcare efficiency. Stratified approaches aim to match patients to the most appropriate care pathways on the basis of their presentation. A common approach bases stratification on patients’ prognostic profile, which requires early, accurate screening using a valid and reliable instrument. By so doing, care decisions aim to offer treatment to those who need it most and avoid over-treatment of those who need it least.

Better matching of patients to care is particularly important for a condition as burdensome as low back pain (LBP) [4, 5]. The prognosis of chronic LBP – when symptoms persist beyond 3 months – is poor [6]. This warrants a focus on the potential for intervention to be appropriately targeted prior to the development of chronic pain. Improved understanding of factors associated with chronic LBP [7–10] has led to the development of self-report questionnaires containing multiple variables known to have prognostic relevance. These prognostic screening instruments (PSIs; also referred to as predictive tools) assess certain characteristics of an individual’s pain experience (including pain intensity and functional impairment) and certain psychosocial factors (e.g. beliefs, catastrophisation, anxiety and depression). These prognostic variables have been shown to be associated with specific outcome measures and time frames [11].

PSIs are widely recommended to inform the management of LBP [12–15], with updated international guidelines encouraging the use of risk stratification to guide care decisions. A possible consequence of these broad recommendations is that PSIs are likely to be used for purposes other than the specific purpose for which they were intended and in varied clinical settings. These factors may impact instrument performance, with implications for care decisions based on screening.

As the use of PSIs to inform care delivery becomes more widely adopted, it is important to further consider the uncertainty that surrounds their accuracy [16, 17]. We investigate how PSIs perform (individually and generally) when administered for the purpose of predicting the likely course of LBP. The aim of this review was to determine how well LBP PSIs discriminate between patients who develop a poor outcome and those who do not in adults with LBP of less than 3 months duration.

## Methods

This systematic review is reported in accordance with the statement for Preferred Reporting Items for Systematic Reviews and Meta-Analysis (PRISMA) [18] (see Additional file 1).

### Registration

Our protocol was registered a priori on the PROSPERO International prospective register of systematic reviews (http://www.crd.york.ac.uk/PROSPERO/display_record.asp?ID=CRD42015015778)

### Data sources and searches

Between June 23 and July 7, 2014, eight electronic databases (Medline (OvidSP), CINAHL (EBSCO host), EMBASE (OvidSP), PsycINFO (OvidSP), PEDro, Cochrane Central Register of Controlled Trials (CENTRAL) (OvidSP), Web of Science (ISI) and SciVerse SCOPUS) were systematically searched by a single reviewer to identify eligible studies. No time limits were applied, but studies were limited to English language publications and those involving human participants. Search terms included the following keywords and their variations: low back pain, sciatica, radiculopathy, risk, screening, questionnaire, instrument, prediction, prognosis, validity. While LBP was of principle interest, studies were not excluded if they involved participants with leg pain/sciatica or radiculopathy (conditions which involve a low-back disorder and are usually accompanied by LBP). Table 1 shows the full search strategy. The reference lists of all included articles and relevant review articles were later searched to identify any additional studies. Searching of all databases was updated on June 29 and December 22, 2015, and June 30, 2016.

**Table 1:** Search Strategy Example. The search strategy below was used to conduct the MEDLINE search for the current systematic review and meta-analysis. Database: Ovid MEDLINE(R) 1946 to Present

#	Searches
1	Back Pain/
2	Low Back Pain/
3	Sciatica/
4	Radiculopathy/
5	(back pain or low back pain or radiculopathy or sciatica or back?ache or lumbago).mp^a^
6	(pain or ache or aching or complaint or dysfunction or disability or disorder).mp^a^
7	Back or spine or lumbar or lumbar spine or low*back).mp^a^
8	6 and 7
9	1 or 2 or 3 or 4 or 5 or 8
10	(screen* or risk screen* or risk).mp^a^
11	(tool or questionnaire or instrument).mp^a^
12	10 and 11
13	9 and 12
14	(predict* or prognosis or prediction rule* or early identification or predictive validity or predictive factors or prognostic or prognostic indicators).mp^a^
15	13 and 14
16	Limit 15 to (English language and humans)

^a^mp: title, abstract, original title, name of substance word, subject heading word, keyword heading word, protocol supplementary concept word, rare disease supplementary concept word, unique identifier

### Eligibility criteria

#### Types of participants

Studies were eligible if they involved adults (aged 18 or over) with ‘recent onset’ LBP (i.e. acute LBP (0–6 weeks) or subacute LBP (6 weeks to 3 months)), with or without leg pain. Studies involving participants with recent-onset and participants with chronic symptoms were included with the intention of requesting from study authors the data from the ‘recent onset’ participants only. Studies including participants with pain in other body regions were considered eligible if more than 75% had LBP. Cohorts of compensable and non-compensable patients presenting to primary, secondary and tertiary care settings were eligible for inclusion. It was also considered appropriate to include individuals registered on workers compensation databases, because it was assumed that this occurs in conjunction with presentation to a healthcare provider. Participants may have presented with a first episode of pain or report episodic/recurrent LBP, provided that the current painful episode was immediately preceded by a minimum of one pain-free month as suggested previously [19].

#### Types of studies

Prospective cohort studies meeting a Level I or Level II quality standard according to the National Health and Medical Research Council of Australia (NHMRC) evidence hierarchy for prognostic studies [20] were included. According to this standard, participants in these studies must have been recruited as a consecutive series of new presentations in any healthcare setting and been subject to longitudinal assessment. Studies classified as NHMRC Level III and IV evidence, including retrospective cohort studies, analysis of a single arm of a randomised controlled trial or case series reports, were excluded. Included studies involved the application of a previously developed PSI within the first 3 months of an episode of LBP and reported follow-up outcomes at a minimum of 12 weeks from initial screening.

We defined a PSI as an instrument that met all of the following criteria: (1) a self-report questionnaire; (2) assesses multiple factors or constructs that have predictive validity for patients with musculoskeletal pain; and (3) was developed to provide prognostic information for musculoskeletal conditions. The broad term of ‘musculoskeletal’ pain rather than LBP was selected to define the PSIs to avoid exclusion of instruments that had been developed for use with musculoskeletal conditions and subsequently validated for LBP cohorts. Studies were not excluded on the basis of how the instrument was developed, or the primary intention of the instrument (ascribed by the developers). For example, the Keele STarT Back Tool (SBT) was developed to include only ‘modifiable’ prognostic factors and was specifically intended for the purpose of matching subgroups of patients to stratified care pathways. Of primary importance to us was the inclusion of all instruments currently being widely used to offer prognostic information, or considered by the wider community of clinicians and researchers to be able to offer prognostic information. Included studies were required to report associations between the PSI scores and participant outcomes, and aimed, a priori, to evaluate the instrument for its predictive validity. Development studies were excluded to avoid including PSIs that had been insufficiently validated for clinical application [21].

#### Types of outcomes

To be included, studies must have reported one or more of the following outcomes:

1. Pain intensity as measured using a visual analogue scale, numeric rating scale (NRS), verbal rating scale or Likert scale
2. Disability as measured by validated self-report questionnaires
3. Sick leave or days absent from work or return to work status
4. Self-reported recovery using a global perceived effect scale or a Likert (recovery) scale

### Study selection

Following removal of duplicate articles, two reviewers independently assessed the titles and abstracts of studies identified by the search for eligibility. AW assessed all the articles; EK and LG each assessed 50% of the articles. All reviewers applied a checklist of inclusion and exclusion criteria. Disagreements were discussed. The full paper was obtained for further assessment if necessary. Full texts of studies potentially fulfilling the eligibility criteria were retrieved, with subsequent independent assessment of all articles undertaken by EK and LG. Reasons for study exclusion were noted on a checklist with any disagreements resolved by discussion.

### Data extraction and analysis

EK and either LG or LR independently reviewed the full text of eligible studies and extracted relevant data using a standardised spreadsheet. Extracted data included details of the healthcare setting, recruitment, study population, number of participants, loss to follow-up, symptom duration, LBP history, compensability, concomitant treatments, outcome measurement, statistical analyses, and reporting quality. Discrepancies in extracted data were identified and checked. If the required data could not be extracted, authors were emailed with the specific enquiry. If no response was received, authors were re-emailed after 2 weeks, and (finally) after a further week.

Predictive validity is conventionally assessed using receiver operating characteristic (ROC) curve analysis, with area under the curve (AUC) statistic being the most routinely reported measure of performance [22]. AUC values provide an overall measure of the discriminative ability of the instrument. Values range from 0.5 to 1.0, where 0.5 indicates that the instrument is no better than chance at discriminating those participants who will have a poor outcome, from those who will recover. AUC values of < 0.6 suggest that the instrument or screening test should be regarded as ‘uninformative’; 0.6–0.7 indicates ‘poor’ discrimination; 0.7–0.8 ‘acceptable’; 0.8–0.9 ‘excellent’; and above 0.9 ‘outstanding’ [23, 24].

Where possible, we extracted AUC values with 95% confidence intervals to enable analysis and comparison of the PSIs. When AUC values were not provided, study authors were requested to either (1) calculate AUC values for the recent-onset participants or (2) provide primary data to allow calculation of AUC values. If the authors chose to calculate AUC values, we offered further instruction on how to do so. The primary outcome of this study was pain intensity at follow-up; poor outcome was pain ≥ 3 on an 11-point NRS, which was based on Grotle et al. [25] and Traeger et al. [26], and follows evidence that many people with scores of < 3 consider themselves to be recovered [27]. All study authors who reported obtaining pain NRS scores were requested to dichotomise pain outcomes according to this definition. Authors then re-analysed their results or offered outcome data and baseline screening scores to enable us to undertake ROC analysis. When authors were willing to assist with dichotomising disability outcomes, scores of ≥ 30% disabled (on their chosen disability outcome measure) were classified as ‘poor outcome’. A similar approach to revision of the ROC analyses was undertaken. No attempt was made to request re-definition of sick leave and recovery outcomes (secondary outcomes of this study).

Meta-analysis was planned considering the potential to pool data according to (1) individual PSIs and (2) specific outcomes. For data pooling to be appropriate, it was considered important that (1) outcome measures were defined consistently, (2) the clinical settings were similar (e.g. all primary care), and (3) uniform statistical analyses had been applied. Interpretation of random effects models was planned due to assumed variability in participant cohorts. Meta-analyses, including tests for statistical heterogeneity (using *I* ^2^ test) were undertaken using MedCalc Statistical Software (version 14.12.0). A post-hoc sensitivity analysis was undertaken to explore the influence of study variation in classification of poor disability outcomes on the meta-analysis.

### Assessment of methodological quality

EK and either LG or LR independently undertook the risk of bias (ROB) assessment using the Quality in Prognostic Studies (QUIPS) tool [28]. This tool was developed specifically for assessing bias in studies of prognostic factors. Items across six domains (study participation, study attrition, prognostic factor measurement, outcome measurement, study confounding, and statistical analysis and reporting) were considered individually for each study. A guideline was used to classify each item as ‘high’, ‘moderate’ or ‘low’ risk of bias. If insufficient information was available to assess potential bias, that domain was rated ‘unclear’. An overall ROB was established for each individual study according to Bruls et al. [29]. The overall ROB for a study was rated as ‘low’ (indicating a high quality study) when all or most (4–6) of the six bias domains were fulfilled, with each domain rated as ‘low’ or ‘moderate’. The overall ROB was rated as ‘high’ (indicating a low quality study) when one or more of the six bias domains were rated as ‘high’ or ‘unclear’. Disagreements in ratings were discussed and, if not resolved, a third reviewer (SH) was consulted. Studies rated as having a ‘low’ risk of bias using the QUIPS tool were considered ‘high quality’.

## Results

### Study selection

Our initial search identified 1557 studies for potential inclusion, from which 110 full text articles were retrieved. Twenty-one studies satisfied all criteria for inclusion. Three further studies were identified through repeat searching. The authors of 13 studies were contacted to request data pertaining specifically to the recent onset participants. Unsuccessful attempts to obtain these data meant that six studies were excluded [30–35]. Eighteen studies were finally included in this review.

**Fig. 1:**
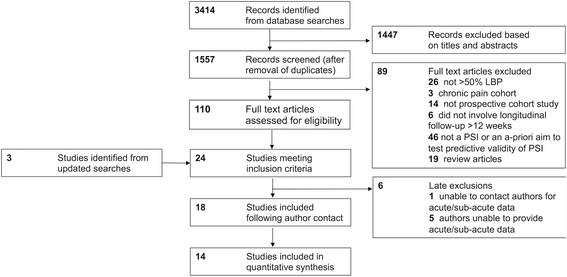
Flow diagram of study selection. *LBP* low back pain, *PSI *prognostic screening instrument

Details of studies accepted and rejected during the selection process are illustrated in Fig. 1. Table 2 details the studies that were excluded based on the participants’ pain duration at baseline screening. Key study characteristics and results are summarised in Table 3 (at the end of the manuscript).

**Table 2:** Studies excluded based on participants pain duration at baseline screening

Reference	Prognostic screening instruments	Reason for exclusion
Bergstrom et al. (2011) [62]	MPI-S	Mixed cohort;^b^ authors did not differentiate an acute/subacute group
Bernstein et al. (1994) [63]	SCL-90-R	Chronic pain cohort (pain > 3 months)
Morso et al. (2011) [64]	PainDETECT questionnaire	Chronic pain cohort (pain duration 3–12 months)
Late exclusions:^a^		
Fischer et al. (2014) [30]	HKF-R10	Mixed cohort;^b^ authors did not differentiate an acute/subacute group
Hurley et al. (2001) [31]	ALBPSQ	Mixed cohort^b,c^
Linton et al. (2011) [32]	OMPSQ (Short Form)	Mixed cohort^b,c^
Morso et al. (2013) [65]	SBT	Mixed cohort^b,c^
Morso et al. (2014) [33]	SBT	Mixed cohort^b,c^
Cats-Baril et al. (1991) [35]	VDPQ	Mixed cohort;^b^ unable to contact authors to request data from recent onset participants

^a^Study authors were contacted (or contact attempts were made) prior to study exclusion

^b^Combination of acute/subacute/chronic pain participants

^c^Authors unable to provide data for ‘recent-onset’ participants

*MPI-S* Multidimensional Pain Inventory (Swedish version), *SCL-90-R* Symptom Checklist 90 Revised, *HKF-R10* Heidelberg Short Early Risk Assessment Questionnaire, *ALBPSQ* Acute Low Back Pain Screening Questionnaire, *OMPSQ* Orebro Musculoskeletal Pain Screening Questionnaire, *SBT* STarT Back Screening Tool, *VDPQ* Vermont Disability Prediction Questionnaire

**Table 3:** Key study characteristics and results

Reference	Country of investigation and clinical setting	Definition of poor outcome	*N* at baseline, (*n* at follow-up, % at follow-up)	Discrimination (AUC (95% confidence interval))
STarT Back Screening Tool				
Beneciuk et al. 2012 [43]	USA Outpatient physiotherapy clinics	At 6 months: ^a^Pain NRS score ≥ 3 ^a^Disability (ODI score ≥ 30%)	73 (55, 75.3%)	^a^Pain 0.61 (0.45–0.76) ^a^Disability 0.75 (0.60–0.90)
Field & Newell, 2012 [44]	UK Chiropractic clinics	At 90 days: ^a^Pain NRS score ≥ 3	477 (151, 31.7%)	^a^Pain 0.597 (0.499–0.694)
Hill et al. 2008 [46]	UK General practice clinics	6 months: RMDQ score ≥ 7 ^a^Pain NRS score ≥ 3 ^a^Disability ≥ 30% RMDQ	177 at follow-up. (*N* at baseline not specified	^a^Pain 0.70 (0.62–0.88) ^a^Disability 0.81 (0.75–0.88)
Kongsted et al. 2015 [38]	Denmark Chiropractic clinics	3 months: ^a^Pain NRS score ≥ 3 ^a^Disability ≥ 30% RMDQ	754 (604, 80.1%)	^a^Pain 0.56 (0.49–0.60) ^a^Disability 0.67 (0.62–0.73)
Newell et al. 2014 [45]	UK Chiropractic clinics	At 90 days: ^a^Pain NRS score ≥ 3	284 (192, 67.6%)	^a^Pain 0.59 (0.48–0.69)
Orebro Musculoskeletal Pain Screening Questionnaire; Acute Low Back Pain Screening Questionnaire				
Gabel et al. 2011 [39]	Australia Physiotherapy outpatient clinics	At 6 months: Functional status ≥ 10% Problem severity > 1 Absenteeism > 0 days Long term absenteeism > 28 days ^a^Pain NRS score ≥ 3 ^a^Disability (SFI score ≥ 30%)	66 (58, 87.9%) (OMPSQ - Original)	Functional status 0.88 (0.78–0.99) Problem severity 0.85 (0.72–0.97) Absenteeism 0.86 (0.76–0.96) Long-term absenteeism 0.85 (0.73–0.96) ^a^Pain 0.84 (0.71–0.97) ^a^Disability 0.80 (0.67–0.92)
Grotle et al. 2006 [25]	Norway General practitioner/Chiropractor/Physiotherapy clinics (27% recruited through advertisement)	At 6 & 12 months: Pain NRS score ≥ 3 Disability (RMDQ score > 4) Sick leave (> 30 days)	123 (112, 91.1%)	Pain 0.62 (0.51–0.73) Disability 0.68 (0.56–0.80) Sick leave 0.80 (0.66–0.93)
Heneweer et al. 2007 [66]	Netherlands Physiotherapy clinics	Not recovered at 12 weeks ^a^Pain NRS score ≥ 3 ^a^Disability QBPDS ≥ 30%)	66 (56, 84.8%)	Non-recovery 0.64 (0.5–0.79) ^a^Pain 0.64 (0.50–0.78) ^a^Disability 0.67 (0.54–0.8)
Jellema et al. 2007 [52]	Netherlands General practice clinics	12 months: score of ‘slightly improved’ or worse at two or more follow-up time points	314 (296, 94.3%)	Non-recovery 0.61 (0.54–0.67)
Law et al. 2013 [37]	China Physiotherapy clinics in public hospitals	12 months post discharge: Failure to return to work Prolonged sick leave (> 30 days)	241 (220, 91.3%)	Return to work 0.69 (0.62–0.76) Prolonged sick leave 0.71 (0.64–0.78)
Nonclercq et al. 2012 [42]	Belgium Emergency facility or outpatient clinic	At 6 months: Pain index score > 16 ODI ≥ 20% Functional index < 45 Work absence > 30 days ^a^Pain NRS score ≥ 3 ^a^Disability ≥ 30% ODI	91 (73, 80%)	Pain 0.73 (no confidence intervals) Functional index 0.79 (no confidence intervals) Absenteeism 0.83 (standard error 0.71) Disability 0.75 (no confidence intervals) ^a^Pain 0.70 (standard error 0.66) ^a^Disability 0.72 (standard error 0.86)
Schmidt et al. 2016 [48]	Germany General practice clinics	6 months: Disability ≥ 4/11 (dichotomised mean response to three GCPS disability items)	181 (112, 62%)	Disability (OMPSQ scale sum score) 0.79 (0.67–0.90) Disability (OMPSQ item sum score) 0.77 (0.66–0.87)
Vermont Disability Prediction Questionnaire				
Hazard et al. 1996 [49]	USA Vermont Department of Labour and Industry database	Not returned to work at 3 months	166 (163, 98%)	Return to work 0.92 (no confidence interval or standard error reported)
Hazard et al. 1997 [50]	USA Vermont Department of Labour and Industry database	Not returned to work at 3 months	304 (268, 88.2%)	Return to work 0.78 (no confidence interval or standard error reported)
Absenteeism Screening Questionnaire				
Truchon et al. 2012 [51]	Canada Quebec Workers Compensation Board database	12 months: Absenteeism > 182 cumulative days	535 (310, 58%)	Absenteeism 0.73 (no confidence intervals or standard error reported)
Chronic Pain Risk Score				
Turner et al. 2013 [61]	USA Primary care	4 months Pain grades 3 & 4 ^a^Pain NRS ≥ 3	458 (425, 92.8%)	Pain grades 3 & 4 0.67 (0.59–0.72) ^a^Pain 0.67 (0.59–0.72)
Back Disability Risk Questionnaire				
Shaw et al. 2009 [40]	USA Occupational health clinics	3 months: Pain ≥ 5 Disability ≥ 50% ^a^Pain NRS score ≥ 3 ^a^Disability ≥ 30% RMDQ	568 (519, 91.4%)	^a^Pain 0.61 (0.56–0.66) ^a^Disability 0.66 (0.62–0.70)
Hancock Clinical Prediction Rule				
Williams et al. 2014 [41]	Australia General practice clinics, Pharmacists or physiotherapy clinics	3 months: No sustained recovery (0 or 1/10 on a NRS for 7 consecutive days) ^a^Pain NRS ≥ 3	956 (937, 82%)	Sustained recovery 0.60 (0.56–0.64) ^a^Pain 0.62 (0.60–0.65)

^a^Unpublished data for ‘recent onset’ participants, provided on request

*NRS* numeric rating scale, *ODI* Oswestry Disability Index, *RMDQ* Roland Morris Disability Questionnaire, *SFI* Spine Functional Index, *QBPDS* Quebec Back Pain Disability Scale, *GCPS* Graded Chronic Pain Scale, *OMPSQ* Orebro Musculoskeletal Pain Screening Questionnaire

### Study characteristics

Included studies were conducted between 1996 and 2015, in 10 different countries – USA (*n* = 5), UK (*n* = 3), Australia (*n* = 2), Netherlands (*n* = 2), and one in each of Norway, Denmark, China, Belgium, Germany, and Canada (Table 3). Seventeen studies included in this review were undertaken in primary healthcare settings, defined, according to the World Health Organization Declaration of Alma-Ata (1978), as involving the individual’s “*first level of contact*” with “*promotive, preventive, curative and rehabilitative services*” ([36] p. 2). One investigation [37] was conducted in a Hospital outpatient physiotherapy setting, considered ‘secondary care’. Five studies included ‘working adult’ populations; 13 studies included ‘general adult’ participants (some of whom were employed). Of those 13 studies, three were undertaken in Physiotherapy settings, four in Chiropractic clinics, six in General Practice settings, two in a Hospital emergency/Outpatient department and two in combinations of these healthcare settings.

### PSIs

Seven instruments satisfied our criteria for classification as a PSI: the SBT (five studies), the Orebro Musculoskeletal Pain Screening Questionnaire (OMPSQ; seven studies), the Vermont Disability Prediction Questionnaire (VDPQ; two studies), the Back Disability Risk Questionnaire (BDRQ; one study), the Absenteeism Screening Questionnaire (ASQ; one study), the Chronic Pain Risk Score (CPRS; one study), and the Hancock Clinical Prediction Rule (HCPR; one study). The PSIs are summarised in Table 4.

**Table 4:** Summary of included predictive screening instruments

Instrument	Summary of instrument	Scoring method	Cut-off scores/subgrouping
STarT Back Tool (SBT) [46]	9-item, self-report questionnaire; items screen for predictors of persistent disabling back pain and include radiating leg pain, pain elsewhere, disability (2 items), fear, anxiety, pessimistic patient expectations, low mood and how much the patient is bothered by their pain; all 9-items use a response format of ‘agree’ or ‘disagree’, with exception to the bothersomeness item, which uses a Likert scale.	Two scores are produced – an overall score and a distress (psychosocial) subscale	Total scores of 3 or less = low risk If total score is 4 or more: - Those with psychosocial subscale scores of 3 or less = medium risk - Those with psychosocial subscale scores of 4 or more = high risk
Orebro Musculoskeletal Pain Screening Questionnaire (OMPSQ) [67] and Acute Low Back Pain Screening Questionnaire (ALBPSQ) [68]	25-item, self-report questionnaires; items screen for six factors: self-perceived function, pain experience, fear-avoidance beliefs, distress, return to work expectancy, and pain coping	Total score calculated from 21 items and can range from 2 to 210 points; higher values indicate more psychosocial problems	A cut-off of 105 proposed for indicating those ‘at risk’ of persisting problems
OMPSQ (Short form) [32]	10-item questionnaire covering five domains: self-perceived function, pain experience, fear-avoidance beliefs, distress, and return to work expectancy; demonstrated to have similar discriminative ability to original OMPSQ	Scores range from 0 to 100 (higher scores indicate higher risk)	A cut-off of 50 recommended to indicate those ‘at risk’ of persisting pain related disability
Vermont Disability Prediction Questionnaire (VDPQ) [49]	11-item self-report questionnaire; assesses perceptions of who was to blame for the injury, relationships with co-workers and employer, confidence that they will be working in 6 months, current work status, job demands, availability of job modifications, length of time employed, and job satisfaction	Hand scored (maximum score of 23)	No optimal cut-off recommended
Back Disability Risk Questionnaire (BDRQ) [40]	16-item self-report questionnaire; items include demographics, health ratings, workplace concerns, pain severity, mood, and expectations for recovery	Sum score calculated	No optimal cut-off recommended
Absenteeism screening questionnaire (ASQ) [51]	16-item, self-report questionnaire; assesses potential occupational back pain disability and risk factors including: work factors (3), physical health (2), supervisor response (1), pain (2), mood (2), wellness/job satisfaction (3), and expectations for recovery (1); mixture of nominal, ordinal and interval scale response options	‘Flag’ related items are summed and level of risk categorised as low, medium or high	0–1 flag items = low risk 2–3 items = medium risk 4–9 items = high risk
Chronic Pain Risk Score (CPRS) [61]	Three graded chronic pain scale ratings of pain intensity, three ratings of activity interference, the number of activity limitation days, the number of days with pain in the past 6 months, depressive symptoms, the number of painful sites	Maximum score of 28 (higher scores indicate greater risk)	No optimal cut-off recommended
Hancock Clinical Prediction Rule (HCPR) [69]	3-item self-report questionnaire, items assess baseline pain (≤ 7/10), pain duration (≤ 5 days) and number of previous painful episodes (≤ 1)	Status on the prediction rule determined by calculating the number of predictors of recovery present	Risk classification based on the number of predictors of recovery present (0–3)

### Outcomes

Six studies assessed pain intensity (using a NRS) as a primary outcome and a further eight studies assessed pain as a secondary outcome. Measures of work absenteeism or self-reported recovery ratings were reported as primary outcomes in four studies each. Disability was assessed as a primary outcome in five studies and as a secondary outcome in a further five studies. Definitions of ‘poor outcome’ (after an episode of LBP) were highly variable. For studies identifying pain as the primary outcome, poor outcome was variably defined as NRS scores of > 0 [38], > 1 [39], > 2 [25], and > 4 [40]; one study [41] defined sustained recovery from LBP by NRS scores of 0 or 1 for 7 consecutive days; one study [42] used a composite pain index.

### Meta-analysis

#### SBT

##### Discrimination of pain outcomes

The five studies [38, 43–46] investigating the SBT used pain as an outcome measure. All authors provided raw data for statistical analysis or followed guidance for analysis of their recent onset data. Consistent classification of ‘poor outcome’ allowed pooling of AUC values (pooled AUC = 0.59 (0.55–0.63); Table 5). Discriminative performance was ‘non-informative’. There was no evidence of statistical heterogeneity (*I* ^2^ = 0.00%, *P* = 0.47).

**Table 5:** Meta-analyses: pooled data specific to predictive screening instrument and outcome measures

PSI	Outcome	Studies (Total N)	Heterogeneity *I* ^2^ (*P*)	Pooled AUC value	95% confidence interval
SBT	Pain (≥ 3)	5 studies (1153)	0.00% (0.47)	0.59	0.55–0.63
SBT	Disability (≥ 30%)	3 studies (821)	80.95% (0.01)	0.74	0.66–0.82
OMPSQ	Pain (≥ 3)	4 studies (360)	40.95% (0.17)	0.69	0.62–0.76
OMPSQ	Disability (≥ 30%)	3 studies (512)	0.00% (0.42)	0.75	0.69–0.82
OMPSQ	6 month absenteeism (> 28 days)	3 studies (243)	0.00% (0.86)	0.83	0.75–0.90
OMPSQ	12 month absenteeism (> 30 days)	2 studies (440)	0.00% (0.90)	0.71	0.64–0.78

*AUC* Area Under the Curve, *SBT* STarT Back Tool, *OMPSQ* Orebro Musculoskeletal Pain Screening Questionnaire

##### Discrimination of disability outcomes

Three SBT studies [38, 43, 46] included disability as an outcome measure. ‘Poor outcome’ (in disability terms) was defined consistently. The pooled AUC value of 0.74 (0.66–0.82) indicated ‘acceptable’ [23, 24] discrimination. There was substantial statistical heterogeneity (*I* ^2^ = 80.95%, *P* = 0.005). To explore the source of heterogeneity, two studies [38, 46] that did not have overlapping confidence intervals were separately removed. Heterogeneity was no longer significant in both analyses (*P* > 0.05), with impact on the AUC values (Table 6).

**Table 6:** Post-hoc sensitivity analysis to explore heterogeneity in STarT Back Screening Tool studies

	AUC	95% Confidence interval	*I* ^2^ (*P*)
All studies included	0.74	0.66–0.82	80.85% (0.01)
Hill et al. (2008) [46] removed	0.68	0.63–0.73	0.00% (0.37)
Kongsted et al. (2015) [38] removed	0.80	0.74–0.86	0.00% (0.42)

*AUC* Area Under the Curve

#### OMPSQ

##### Discrimination of pain outcomes

Four of the seven studies [25, 39, 42, 47] investigating the OMPSQ included pain as an outcome measure. Consistent classification of ‘poor outcome’ was achieved, allowing pooling of all AUC values (pooled AUC = 0.69 (0.62–0.76); Table 5). Discriminative performance was ‘poor’. Statistical heterogeneity was moderate but not statistically significant (*I* ^2^ = 40.95%, *P* = 0.17).

##### Discrimination of disability outcomes

Five OMPSQ studies included disability as an outcome measure. Three studies classified ‘poor outcome’ as ≥ 30% disability [39, 42, 47], one used ≥ 20% [25] and one used ≥ 40% [48]. Despite different definitions, the results were pooled and post-hoc sensitivity analysis confirmed this to be acceptable (Table 7). Discriminative performance was ‘acceptable’ [23, 24] (pooled AUC = 0.75 (0.69–0.82)). There was no evidence of statistical heterogeneity (*I* ^2^ = 0.00%, *P* = 0.64).

**Table 7:** Post-hoc sensitivity analysis to explore the effect of poor outcome classification on the discriminative performance of the Orebro Musculoskeletal Pain Screening Questionnaire

	AUC	95% Confidence interval	*I* ^2^ (*P*)
All studies included	0.75	0.69–0.82	0.00% (0.64)
Schmidt et al. (2016) [48] removed (≥ 40%)	0.73	0.65–0.81	0.00% (0.60)
Grotle et al. (2006) [25] removed (≥ 20%)	0.75	0.69–0.82	0.00% (0.50)
Schmidt et al. (2016) [48] and Grotle et al. (2006) removed [25]	0.74	0.65–0.82	0.00% (0.42)

*AUC* Area Under the Curve

##### Discrimination of absenteeism outcomes

The OMPSQ offers ‘excellent’ discrimination of prolonged absenteeism at 6 months (pooled AUC from three studies [25, 39, 42] = 0.83 (0.75–0.90); and ‘acceptable’ discrimination of prolonged absenteeism at 12 months (pooled AUC from two studies [25, 37] = 0.71 (0.64–0.78). There was no statistical heterogeneity (*I* ^2^ = 0.00%, *P* = 0.86).

#### All instruments

##### Discrimination of pain outcomes

**Fig. 2:**
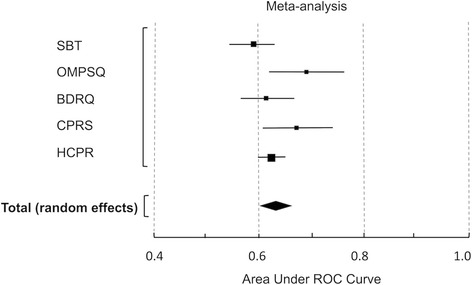
Meta-analysis of the discriminative performance of all instruments (for pain). *SBT* STarT Back Tool, *OMPSQ* Orebro Musculoskeletal Pain Screening Questionnaire, *BDRQ* Back Disability Risk Quesionnaire, *CPRS* Chronic Pain Risk Score, *HCPR* Hancock Clinical Prediction Rule, *ROC* Receiver Operating Characteristic

Twelve investigations in primary care settings (using five different PSIs) reported pain outcomes at medium term follow-up. Poor outcome was consistently defined as NRS scores ≥ 3. Data were pooled for studies using the SBT and OMPSQ. Meta-analysis enabled visual comparison of the discriminative performances of all instruments (Fig. 2). The pooled performance was ‘poor’ (pooled AUC = 0.63 (0.60–0.65)). The *I* ^2^ of 51.16% may represent moderate statistical heterogeneity (*P* = 0.08).

##### Discrimination of disability outcomes

Nine studies (involving three PSIs) reported disability outcomes at medium term follow-up. Poor outcome was consistently defined as ≥ 30% disabled, with the exception of two of the OMPSQ studies as noted previously (Grotle et al. [25] ≥ 20% and Schmidt et al. [48] ≥ 40%).

**Fig. 3:**
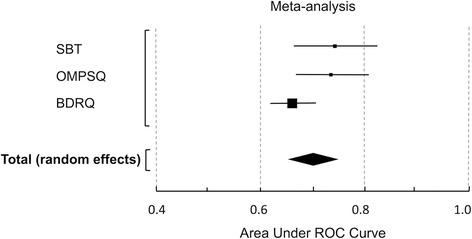
Meta-analysis of the discriminative performance of all instruments (for disability). *SBT* STarT Back Tool, *OMPSQ* Orebro Musculoskeletal Pain Screening Questionnaire, *BDRQ* Back Disability Risk Quesionnaire, *ROC* Receiver Operating Characteristic

Data were pooled for studies using the SBT and the OMPSQ. Meta-analysis enabled visual comparison of the discriminative performances of all instruments (Fig. 3). The pooled performance was ‘acceptable’ (pooled AUC = 0.71 (0.66–0.76)) and indicated substantial heterogeneity (*I* ^2^ = 69.89%, *P* = 0.04). Graphical representation suggests that the SBT and the OMPSQ out-performed the BDRQ. Heterogeneity was resolved with removal of the BDRQ study: pooled AUC = 0.75 (0.70–0.80, *I* ^2^ = 0.00%, *P* = 0.98).

##### Discrimination of absenteeism outcomes

Variability in follow-up time-points and outcome measures used in studies with absenteeism outcomes [25, 39, 40, 42, 49–51] did not allow comparisons to be made between instruments.

### Studies not included in the meta-analysis

The following four of studies were not included in a quantitative meta-analysis since they used outcome measures dissimilar to the measures used in the other included studies.

#### Jellema et al. 2007 [52] – OMPSQ

This study investigated the use of the OMPSQ in a general adult population for prediction of non-recovery at 12 months post-screening (defined as a score of slightly improved or worse on a 7-point Likert scale, at two or more follow-up time points). ‘Good’ instrument calibration was reported (i.e. agreement between predicted and observed risks); however, discriminative ability for predicting long-term global recovery was poor (AUC = 0.61 (0.54–0.67).

#### Hazard et al. 1996 [49] & 1997 [50] – VDPQ

These studies of prognostic screening indicated the potential utility of the VDPQ to predict return to work at 3 months post low back injury. The initial validation study [49] revealed ‘outstanding’ discriminative performance (AUC = 0.92, no confidence intervals obtained) and the subsequent study [50] suggested it was ‘acceptable’ (AUC = 0.78; no confidence intervals obtained).

#### Truchon et al. (2012) [51] – ASQ

This study suggested ‘acceptable’ discrimination of long-term absenteeism (>182 cumulative days) at 12-month follow-up using the ASQ (AUC = 0.73; no confidence intervals obtained).

### Methodologic quality

Sixteen of the 18 included studies were assessed to have a low risk of bias and were thereby regarded to be of high quality. Two studies were regarded to have a high risk of bias primarily due to a high rate of loss to follow-up (> 40%). The assessment of individual study quality is reported in Table 8 (at the end of the manuscript).

**Table 8:** Methodological assessment of included studies

Study	A. Study participation	B. Study attrition	C. Prognostic factor measurement	D. Outcome measurement	E. Study confounding	F. Statistical analysis and reporting	Overall assessment of risk of bias^a^
Beneciuk et al. 2012 [43]	Low	Moderate	Moderate	Low	Low	Low	Low
Field & Newell 2012 [44]	Moderate	Moderate	Low	Low	Low	Low	Low
Gabel et al. 2011 [39]	Moderate	Low	Moderate	Low	Low	Low	Low
Grotle et al. 2006 [25]	Moderate	Low	Moderate	Low	Low	Moderate	Low
Hazard et al. 1996 [49]	Moderate	Low	Low	Low	Low	Moderate	Low
Hazard et al. 1997 [50]	Moderate	Low	Low	Low	Low	Low	Low
Heneweer et al. 2007 [66]	Moderate	Low	Low	Low	Low	Low	Low
Hill et al. 2008 [46]	Moderate	Moderate	Low	Low	Low	Low	Low
Jellema et al. 2007 [52]	Low	Low	Low	Moderate	Low	Low	Low
Kongsted et al. 2015 [38]	Low	Low	Low	Low	Low	Low	Low
Law et al. 2013 [37]	Low	Moderate	Low	Low	Moderate	Low	Low
Newell et al. 2014 [45]	Low	High	Moderate	Low	Low	Low	High
Nonclercq et al. 2010 [42]	Moderate	Low	Low	Low	Low	Low	Low
Shaw et al. 2009 [40]	Low	Low	Low	Low	Low	Low	Low
Schmidt et al. 2016 [48]	Moderate	Moderate	Low	Low	Low	Low	Low
Truchon et al. 2012 [51]	Moderate	High	Low	Moderate	Low	Moderate	High
Turner et al. 2013 [61]	Moderate	Low	Low	Low	Low	Low	Low
Williams et al. 2014 [41]	Low	Low	Low	Low	Low	Low	Low

^a^The overall assessment of risk of bias for a study was rated as ‘low’ when all or most (4–6) of the six bias domains were fulfilled, with each domain rated as ‘low’ or ‘moderate’. The overall risk of bias was rated as ‘high’ when one or more of the six bias domains were rated as ‘high’ or ‘unclear’. Studies with low overall risk of bias were considered high quality

## Discussion

Based on high quality prognostic studies, this systematic review provides evidence that LBP PSIs perform poorly at assigning higher risk scores to individuals who develop chronic pain, than to those who do not. Clinicians can expect that a PSI, administered within the first 3 months of an episode of LBP will correctly classify a patient as high or low risk of developing chronic pain between 60% and 70% of the time. PSIs perform somewhat better at discriminating between patients who will and will not have persisting disability (70–80% probability of correct classification) and appear most successful (> 80% probability) at discriminating between patients who will or will not return to work successfully.

This review also informs about the prognostic performance of specific instruments. The OMPSQ and VDPQ appear to perform well at predicting return to work outcomes and the SBT and the OMPSQ have modest predictive value for disability outcomes, but the included instruments demonstrate little value for informing about likely pain outcomes. Problems associated with using a screening instrument for a purpose other than intended (i.e. based on interest in a specifically defined outcome, at a specific time point) have been introduced in this paper. The instruments included in this study were designed to predict outcomes at time points varying between 3 and 6 months. Two were designed to predict work absenteeism (VDPQ, ASQ), one to predict status on a chronic pain scale (CPRS), one to predict LBP recovery (HCPR), and one to predict functional limitation (SBT). Only two instruments (BDRQ, OMPSQ) were developed to predict more than one clinical outcome. This may have played a role in the poor performance of several of the instruments when evaluated according to the uniform methods we employed.

While our classification of the SBT as a PSI may be arguable, we considered that its clinical use as a prognostic instrument warranted its inclusion in this review. The NICE guidelines [15] recommend that clinicians use tools such as the SBT to identify patients at risk of poor outcome and tailor their management accordingly. Our findings suggest, however, that there is need for caution if the SBT is administered only for the purpose of predicting the risk of poor outcome. As a ‘stratified care tool’ with matched treatment pathways, the merits of the SBT have been reported elsewhere [2, 53].

While it is ideal that stratified care tools such as the SBT have high predictive validity this may not be realistic if the approach is to only include modifiable items during instrument development. Additionally, screening instruments designed for clinical use must be brief and simple to score. A trade-off of these factors may be reduced discriminative performance. It can be noted that the discriminative performance of the SBT is better in a UK General Practice setting than in Physiotherapy or Chiropractic settings – a finding consistent with the understanding that the usefulness of a screening instrument is highly setting-specific [44, 54] and optimal in the cohort for which it was developed [55]. In contrast, however, the ‘excellent’ performance of the OMPSQ for discriminating workers at risk of prolonged absenteeism regardless of country and across varied clinical settings suggests the wider utility of this PSI.

This study was prospectively registered with full adherence to the published protocol. We used the QUIPS methodological appraisal tool [28], a valid and reliable tool for evaluating prognostic studies. The general quality of included studies was assessed to be high with the exception of two studies that had high loss to follow-up [44, 51]. To our knowledge, this is the first quantitative synthesis and analysis of the discriminative performance of PSIs. All previous systematic reviews of PSIs have been unable to conduct meta-analyses of predictive accuracy because of clinical heterogeneity [9, 17, 56, 57]. It is also the first review to include studies testing the SBT. Additional data obtained from study authors facilitated data pooling from similar adult populations, with consistent follow-up time points and identical classifications of poor outcome. Pooling data from instruments that were designed with different purposes in mind may, however, limit the strength of the conclusions that can be drawn from this study.

ROC analyses are recommended for discriminative accuracy studies [58], but come with some limitations. A ROC analysis requires dichotomisation of outcomes, which means that the definition of ‘poor outcome’ can affect findings. In the absence of a general consensus on the definition of ‘poor outcome’, we followed previous studies and recommendations [24, 27, 59]. The selected cut-off score of ≥ 3/10 on a pain NRS was based on the understanding that many people with pain scores of < 3 consider themselves to be ‘recovered’ [1]. Boonstra et al. [60] support that people with pain NRS scores of ≤ 3 describe themselves to be experiencing only ‘mild’ symptoms. We classified participants who were ‘not recovered’ at follow-up (or those experiencing more than mild symptoms) as having a ‘poor outcome’. Since the outcome classification can influence discriminative performance, it would have been interesting to evaluate alternative cut-off points for poor outcome for each of the outcomes considered; this could be considered in further research. The definitions we applied were used by several included studies [25, 39, 42, 61]. In addition, AUC values (derived from the ROC analysis) are a function of sensitivity and specificity – both of which are influenced by cohort characteristics (e.g. symptom severity and psychological profile). Variations are therefore expected for the same instrument among different populations.

Recommendations for the management of LBP in primary care frequently include using available screening instruments to obtain information about ‘risk’ of a poor outcome. This review highlights that clinicians may need be cautious about placing too much weight on PSIs during their clinical assessment, under the misimpression that they are able to accurately determine chronic pain risk. Using PSIs to allocate care carries the risk that patients misclassified by PSIs as low-risk are undertreated and patients misclassified as high-risk are overtreated. Estimation of risk of poor disability outcomes and prolonged absenteeism are likely to be more accurate – indicating that it is necessary to consider the clinical outcomes of interest when seeking prognostic information.

It is important to note, however, that this study investigated the predictive performance of PSIs and does not inform whether the implementation of prognostic screening improves outcomes for adults with recent onset LBP. Alternative research approaches, namely randomised ‘impact’ trials [1], are required to address this question. Furthermore, it is relevant to consider whether the use of PSIs offers more accurate estimation of a patient’s course of LBP than clinician judgement. Previous studies comparing the discriminative performance of screening instruments (including the SBT and the OMPSQ) with primary care clinicians’ estimation of risk of poor outcome [52, 38] have failed to show superior capabilities of the questionnaires.

As highlighted in the PROGRESS recommendations [21], the validation of predictive models requires a succession of steps from development through to external validation and impact analysis – a process which has been only partially fulfilled by the PSIs in this review. Further research according to PROGRESS recommendations will allow improved confidence in the selection and application of available instruments. Less understood factors (e.g. structural pathology, sleep or social factors) should be further investigated and integrated into prognostic models to improve predictive accuracy beyond what is currently achievable. In addition, there remains a need to undertake further prospective clinical trials investigating the effectiveness of screening to direct stratified care approaches for patients with LBP. The performance of a stratified care instrument is best evaluated by an effect size derived from a randomised controlled trial.

## Conclusions

LBP screening instruments administered in primary care perform poorly at assigning higher risk scores to individuals who develop chronic pain, than to those who do not develop chronic pain. Risks of a poor disability outcome and prolonged absenteeism are likely to be estimated with greater accuracy. While PSIs may have useful clinical application, it is important that clinicians who use screening tools to obtain prognostic information consider the potential for misclassification of patient risk and its consequences for care decisions based on screening. However, it needs to be acknowledged that the outcomes on which we evaluated these screening instruments in some cases had a different threshold, outcome and time period than those they were designed to predict.

## Additional file

Additional file 1: PRISMA Checklist. (DOC 64 kb)

## Abbreviations

ASQ: Absenteeism Screening Questionnaire
AUC: area under the curve
BDRQ: Back Disability Risk Questionnaire
CPRS: Chronic Pain Risk Score
HCPR: Hancock Clinical Prediction Rule
LBP: low back pain
NHMRC: National Health and Medical Research Council of Australia
NRS: numeric rating scale
ODI: Oswestry Disability Index
OMPSQ: Orebro Musculoskeletal Pain Screening Questionnaire
PRISMA: Preferred Reporting Items for Systematic reviews and Meta-Analysis
PSI: prognostic screening instrument
QBPDS: Quebec Back Pain Disability Score
QUIPS: QUality In Prognostic Studies
ROB: risk of bias
ROC: receiver operating characteristic
SBT: STarT Back Tool
VDPQ: Vermont Disability Prediction Questionnaire
