# Erratum to: Can screening instruments accurately determine poor outcome risk in adults with recent onset low back pain? A systematic review and meta-analysis

**DOI:** 10.1186/s12916-017-0814-8

**Published:** 2017-02-17

**Authors:** Emma L. Karran, James H. McAuley, Adrian C. Traeger, Susan L. Hillier, Luzia Grabherr, Leslie N. Russek, G. Lorimer Moseley

**Affiliations:** 1 Sansom Institute for Health Research, University of South Australia, GPO Box 2471, Adelaide, South Australia 5001 Australia; 2 Neuroscience Research Australia, Barker Street Randwick, Sydney, New South Wales 2031 Australia; 3 Clarkson University, 41 Elm Street, Potsdam, New York USA; 4 Prince of Wales Clinical School, University of New South Wales, High Street, Kensington, New South Wales 2052 Australia

## Erratum

After publication of the original article [1], it was brought to the authors’ attention that there is an error in Table 4. The Absenteeism Screening Questionnaire (Truchon et al. 2012) has been summarised incorrectly, requiring changes to the Summary of Instruments, Scoring Method and Cut-off scores/sub-grouping fields.

**Table 4:** Summary of included predictive screening instruments

Instrument	Summary of instrument	Scoring method	Cut-off scores/sub-grouping
SBT STarT Back Tool [50]	9 Item, self-report questionnaire. Items screen for predictors of persistent disabling back pain and include radiating leg pain, pain elsewhere, disability (2 items), fear, anxiety, pessimistic patient expectations, low mood and how much the patient is bothered by their pain. All 9-items use a response format of ‘agree’ or ‘disagree’, with exception to the bothersomeness item, which uses a Likert scale.	Two scores are produced: an overall score and a distress (psychosocial) subscale.	Total scores of 3 or less = low risk. If total score is 4 or more: - Those with psychosocial subscale scores of 3 or less = medium risk - Those with psychosocial subscale scores of 4 or more = high risk
OMPSQ Orebro Musculoskeletal Pain Screening Questionnaire [68] & ALBPSQ Acute Low Back Pain Screening Questionnaire [69]	25 item, self-report questionnaires. Items screen for six factors: self-perceived function, pain experience, fear-avoidance beliefs, distress & return to work expectancy and pain coping.	Total score calculated from 21 items and can range from 2 – 210 points. Higher values indicate more psychosocial problems.	A cut-off of 105 proposed for indicating those ‘at risk’ of persisting problems
OMPSQ (Short form) Orebro Musculoskeletal Pain Screening Questionnaire (Short form) [32]	10 Item questionnaire covering 5 domains: self-perceived function, pain experience, fear-avoidance beliefs, distress & return to work expectancy. Demonstrated to have similar discriminative ability to original OMPSQ.	Scores range from 0–100 (higher scores indicate higher risk).	A cut-off of 50 recommended to indicate those ‘at risk’ of persisting pain related disability.
VDPQ Vermont Disability Prediction Questionnaire [53]	11 Item self-report questionnaire. Assesses perceptions of who was to blame for the injury, relationships with co-workers and employer, confidence that he/she will be working in 6 months, current work status, job demands, availability of job modifications, length of time employed, and job satisfaction.	Hand scored (maximum score of 23).	No optimal cut-off recommended.
BDRQ Back Disability Risk Questionnaire [44]	16 Item self-report questionnaire. Items include demographics, health ratings, workplace concerns, pain severity, mood and expectations for recovery.	Sum score calculated.	No optimal cut-off recommended.
ASQ Absenteeism screening questionnaire [55]	22 item, self-report questionnaire. Assesses six sub-sections of variables: fear-avoidance beliefs related to work, return to work expectations, annual family income before-taxes, last level of education attained, work schedule and work concerns.	Total score calculated using scoring template.	No optimal cut-off recommended.
CPRS Chronic Pain Risk Score [65]	3 graded chronic pain scale ratings of pain intensity, 3 ratings of activity interference, the number of activity limitation days, the number of days with pain in the past 6 months, depressive symptoms, the number of painful sites.	Maximum score of 28 (higher scores indicate greater risk).	No optimal cut-off recommended.
HCPR Hancock Clinical Prediction Rule [70]	3 item self-report questionnaire, items assess baseline pain (≤7/10), pain duration (≤5 days) and number of previous painful episodes (≤1).	Status on the prediction rule determined by calculating the number of predictors of recovery present.	Risk classification based on the number of predictors of recovery present (0–3).

The amended version of Table 4 is published in this erratum. The contents of Table 4 in no way impact on the analysis or results of this study, or their interpretation.
